# Lineage relationship between prostate adenocarcinoma and small cell carcinoma

**DOI:** 10.1186/s12885-019-5680-7

**Published:** 2019-05-30

**Authors:** Adelle D. Kanan, Eva Corey, Ricardo Z. N. Vêncio, Arjun Ishwar, Alvin Y. Liu

**Affiliations:** 1 Department of Urology, University of Washington, Box 358056, 850 Republican Street, Seattle, Washington 98195-6100 USA; 2 Institute for Stem Cell and Regenerative Medicine, University of Washington, Seattle, Washington USA; 3 Department of Mathematics, University of Sao Paulo, 3900 Ave Bandeirantes, Vila Monte Alegre, Ribeirão Preto 14040-900 Brazil; 4 Thermo Fisher Scientific, 168 3rd Ave, Waltham, Massachutts 02451 USA; 5 Sophia Genetics, 1550 E Campbell Ave. #4032, Phoenix, Arizona 85014 USA

**Keywords:** Small cell carcinoma, Stem cell factors, Reprogramming, Cancer de-differentiation

## Abstract

**Background:**

Prostate cancer displays different morphologies which, in turn, affect patient outcome. This fact prompted questions about the lineage relationship between differentiated, more treatable prostate adenocarcinoma and poorly differentiated, less treatable non-adenocarcinoma including small cell carcinoma, and the molecular mechanism underlying prostate cancer differentiation.

**Methods:**

Newly available non-adenocarcinoma/small cell carcinoma PDX LuCaP lines were analyzed for expression of stem cell transcription factors (scTF) LIN28A, NANOG, POU5F1, SOX2, which are responsible for reprogramming or de-differentiation. cDNA of these genes were cloned from small cell carcinoma LuCaP 145.1 into expression vectors to determine if they could function in reprogramming.

**Results:**

Expression of scTF was detected in small cell carcinoma LuCaP 93, 145.1, 145.2, and non-adenocarcinoma LuCaP 173.1, 173.2A. Transfection of scTF from LuCaP 145.1 altered the gene expression of prostate non-small cell carcinoma cells, as well as fibroblasts. The resultant cells grew in stem-like colonies. Of note was a 10-fold lower expression of B2M in the transfected cells. Low B2M was also characteristic of LuCaP 145.1. Conversely, B2M was increased when stem cells were induced to differentiate.

**Conclusions:**

This work suggested a pathway in the emergence of non-adenocarcinoma/small cell carcinoma from adenocarcinoma through activation of scTF genes that produced cancer de-differentiation.

**Electronic supplementary material:**

The online version of this article (10.1186/s12885-019-5680-7) contains supplementary material, which is available to authorized users.

## Background

Prostate cancer is a common malignancy in men, and can be treated successfully if the cancer is of low grade. Low-grade tumors are well-differentiated with glandular formation (adenocarcinoma). High-grade tumors are poorly differentiated with no glandular formation (non-adenocarcinoma). Androgen deprivation therapy can be effective when the cancer recurs after initial treatment. However, in many patients undergoing this treatment, the cancer becomes castration resistant. One notable tumor type in these advanced diseases is small cell carcinoma. It is highly aggressive, and does not respond well to anti-cancer agents [1].

How do small cell carcinoma arise? How do cancer cells transition from a well-differentiated morphology to a poorly differentiated one? Relevant to answering these questions is the characterization of prostate cancer cells as either luminal-like (i.e., similar to normal luminal cells with a few hundred differentially expressed genes) or stem-like (i.e., dissimilar to luminal cells with thousands of differentially expressed genes) [2, 3]. The former included mainly adenocarcinoma while the latter non-adenocarcinoma and small cell carcinoma. This dichotomy of cancer cell types was visualized in a principal components analysis (PCA) space generated from the transcriptomes of prostate luminal, basal, stromal, endothelial (differentiated cell types), plus those of stem cells [embryonic stem (ES), embryonal carcinoma (EC), induced pluripotent stem (iPS)] [4]. Since stem cells give rise to somatic cells through differentiation, cancer cell differentiation might also be involved in the generation of multiple cancer cell types. Cancer cell differentiation could proceed from a cancer stem-like cell type to luminal-like adenocarcinoma. This differentiation could be arrested at intermediate stages to produce more stem-like types such as non-adenocarcinoma and small cell carcinoma. Alternatively, stem-like types could arise from de-differentiation as seen in reprogramming of differentiated somatic cells via forced expression of a set of stem cell transcription factors (scTF) [5]. Other researchers suggested that transformation of basal epithelial cells, present in benign glands but not in tumor glands, gave rise to poorly differentiated cancer cells. CD44^+^ CD49f^+^ basal cells were postulated to be the prostate progenitor cells. Transformed basal cells (through in vitro transfection of vectors containing oncogenes) produced highly aggressive cancer cells [6, 7]. However, transcriptomes of prostate cancer cell types analyzed, to date, evinced no expression signature of basal cells [2, 8]. Basal cells express few, if any, stem cell markers. Rather, they represent a differentiated cell type as shown by the different gene expression of basal cells in the prostate and bladder [8, 9].

Previously, we reported the presence of scTF LIN28A, NANOG, POU5F1 and SOX2 in a small cell carcinoma patient-derived xenograft (PDX) line LuCaP 145.1 but absent in adenocarcinoma PDX lines [10]. We chose these four scTF specifically because they can perform reprogramming [11]. In addition, LuCaP 145.1 was found to share expression of many genes with stem cells, including the down-regulation of β2-microglobulin (B2M) [10]. B2M is a so-called housekeeping marker in adult cell types, both normal and cancerous. It is commonly employed as a control in reverse transcriptase-polymerase chain reaction (RT-PCR) analysis of gene expression. More recently established LuCaP including non-adenocarcinoma and small cell carcinoma allowed us to examine scTF expression in lines other than LuCaP 145.1. The > 30 LuCaP lines were established from human tumors and propagated in male SCID mice. Both transcriptomic analysis and immunostaining have shown concordance between LuCaP tumor cells and their corresponding human donor tumor tissues [12–16]. Significantly, we also wanted to determine if the scTF genes in LuCaP 145.1 were responsible for the gene expression of stem-like cancer types. Accordingly, these genes were cloned from LuCaP 145.1 into expression vectors for cell transfection. The goal of this research was to test the hypothesis that adenocarcinoma vs. non-adenocarcinoma/small cell carcinoma are related through cancer de-differentiation.

## Methods

### LuCaP small cell carcinoma PDX lines

In the LuCaP PDX family (tissue origin), non-adenocarcinoma lines were represented by LuCaP 173.1 (liver metastasis), LuCaP 173.2A (rib metastasis), and small cell carcinoma with neuroendocrine features LuCaP 93 (TURP), LuCaP 145.1 (liver metastasis), LuCaP 145.2 (lymph node metastasis). The LuCaP lines were passaged in mice and harvested when the tumors reached 400–800 mg. Tumor pieces weighing 100 mg were minced and digested with collagenase type 1 (ThermoFisher, Waltham, MA) in culture media overnight. The resultant cells were resuspended in Hank’s balanced salt solution and centrifuged in Percoll (GE Healthcare Bio-Sciences, Chicago, IL) density gradients (500 g, 30 min) [17]. In Percoll, cancer cells prepared from human primary prostate tumors band at the epithelial cell density ρ = 1.07 [epi] while prostate stromal cells band at the stromal cell density ρ = 1.035 [strom] [17]. Adenocarcinoma LuCaP lines banded at [epi] as reported previously [10]. Cells of LuCaP tumors banded in Percoll were collected by 18-gauge needle for RNA isolation (Ambion RNAqueous-Micro, ThermoFisher) and cDNA synthesis.

### Gene expression analysis

scTF gene expression in LuCaP cells was analyzed by RT-PCR. The oligonucleotide primer pairs for LIN28A, NANOG, POU5F1 and SOX2 scTF were reported previously [10, 18]. The expected reaction product sizes were 660 bp POU5F1; 570 bp SOX2; 750 bp NANOG; 650 bp LIN28A. Primer pairs for cloning full length scTF cDNA from LuCaP 145.1 into plasmid vector pVITRO1-neo are described in Additional file 1 Primer pairs for expression analysis of h(uman)B2M were CACGTCATCCAGCAGAGAATGGAAAGTC and TGACCAAGATGT.

TGATGTTGGATAAGAG (300-bp product); m(ouse)B2M CTGCTACGTAACACAGTTCCACC and CATGATGCTTGATCACATGTCTC (240-bp product). All primers were synthesized by IDT (Coralville, IA). The PCR conditions used were 35 cycles of 94°, 30s; 57°, 30 s; 72°, 60 s.

### Mammalian cell transfection

Supercoiled (2–5 μL DNA from 1-mL culture resuspended in 50 μL H_2_O) or *Pac*I-digested plasmids pLP4 (LIN28A and POU5F1) and pSN2 (SOX2 and NANOG), shown in Additional file 2, were used to transfect cells harvested from near confluent culture (~ 2 × 10^6^ cells). Human embryonic kidney fibroblasts HEK293F (ThermoFisher), prostate cancer cell lines C4–2B, LNCaP and PC3 were grown in RPMI1640 media supplemented with 10% fetal bovine serum (FBS) = complete media (CM). LNCaP and C4–2B are lineage related [19]. C4–2B was established from orthotopic implantation of C4–2, while C4–2 was derived from LNCaP implanted with bone stromal cells in castrated mice, and subsequent bone metastasis. PC3 was established from a bone metastasis (see ref. [2]). LNCaP and C4–2B are adenocarcinoma-like while PC3 is non-adenocarcinoma-like. After trypsin, the cells were resuspended in 80 μL Electroporation Buffer and 20 μL Supplement 1 (Lonza AMAXA Biosystems, Basel, Switzerland) with pLP4 and pSN2 in electroporation cuvette. The electroporator programs used were A-024 for 293F and S-005 for cancer cells. The shocked cells were withdrawn with 500 μL media and added to 10-cm plates in 8 mL CM. After 3 d, G418 sulfate (Corning Mediatech, Corning, NY) was added to the culture media at 1 mg/mL to select neo^R^ transfected cells. In about a week, discrete G418-resistant colonies of cells were evident. Cloning was achieved by picking the visible colonies with sterile pipetman tips into 6-well plates. The transformed cells were labeled with asterisk: 293F*, LNCaP*, C4–2B*, PC3*.

### Growth of transfected cells

In addition to growth in CM under normoxia, cells were transferred to Matrigel-coated plates, on irradiated mouse embryo fibroblasts (MEF) in serum-free media supplemented with 10% KnockOut Serum Replacement (KSR, ThermoFisher), and grown under hypoxia (5% O_2_) [10, 18]. Cells were processed for RNA isolation and gene expression analysis. Primers for *neo* were GCAGCTGTGCTCGACGTTGTCACTG and CAGAGTCCCGCTCAGAAGAACTCGTC (560-bp product).

### DNA microarray analysis

RNA prepared from clones was analyzed by Human Genome U133 Plus 2.0 GeneChips (Affymetrix, Santa Clara, CA). This particular array was used so that the generated datasets could be compared with those obtained with this array in the past. Cross-platform analysis (e.g., Agilent arrays vs. Affymetrix arrays) was found to be not possible. The array results were normalized with Affymetrix software, and data analysis was described previously [20]. DNA microarray signal intensity values provided a quantitative measure of gene expression, e.g., down- or up-regulation of B2M, to support the RT-PCR results [10].

### Transcriptome dataset query

Cell-type transcriptome datasets archived in our public UESC database (http://scgap.systemsbiology.net/) were queried as described in ref. [21]. Probeset signal intensity values were retrieved and displayed on a gray scale.

## Results

### Expression of LIN28A, NANOG, POU5F1, SOX2 by non-adenocarcinoma LuCaP

**Fig. 1:**
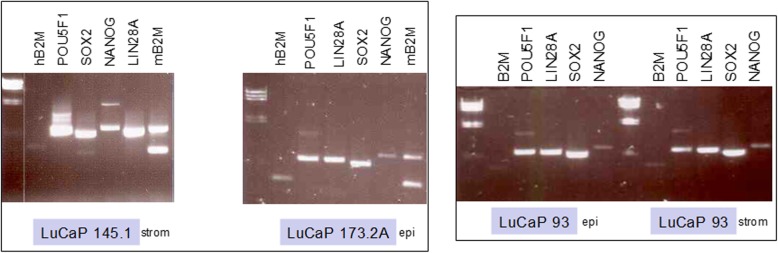
Expression of scTF in LuCaP small cell carcinoma lines. Shown are the RT-PCR results for LuCaP 145.1 [strom], LuCaP 173.2A [epi], LuCaP 93 [epi] and LuCaP 93 [strom]: 650 bp LIN28A, 750 bp NANOG, 660 bp POU5F1, 570 bp SOX2. The mB2M product contained an extra band of larger size. Each gene reaction was done with no cDNA input as control

In Percoll, the bulk of LuCaP 145.1 tumor cells banded at [strom] instead of [epi]. The cells collected at [strom] showed expression of LIN28A, NANOG, POU5F1, SOX2 and low expression of hB2M (Fig. 1). Signals from mB2M indicated co-banded mouse cells (fibroblasts at [strom]) in the harvested xenograft. The scTF signals were not from the mouse cells as any mouse stem cells would unlikely be present in the tumor xenografts. No signals were detected from what was collected at [epi]. A similar pattern was obtained with LuCaP 145.2 (data not shown) established from a different metastasis than LuCaP 145.1 in the same patient donor. The other small cell carcinoma line had cells collected at both densities as shown for LuCaP 93 [strom] and LuCaP 93 [epi]; the bulk of LuCaP 173.2A was collected at [epi] (Fig. 1). Expression in partitioned LuCaP 173.1, established from a different metastasis in the same patient donor, was similar to that of LuCaP 173.2A. Unlike LuCaP 145.1 and 145.2, NANOG expression as judged by the product band intensity was lower in these other LuCaP. Also by band intensity, the level of hB2M was higher in LuCaP 173.2A (a non-small cell carcinoma). The expression levels were in general agreement with signal values of transcriptome analyses of LuCaP lines by RNAseq (E. Corey, unpublished data).

### Functional testing of LuCaP 145.1-derived scTF genes in reprogramming of human fibroblast

**Fig. 2:**
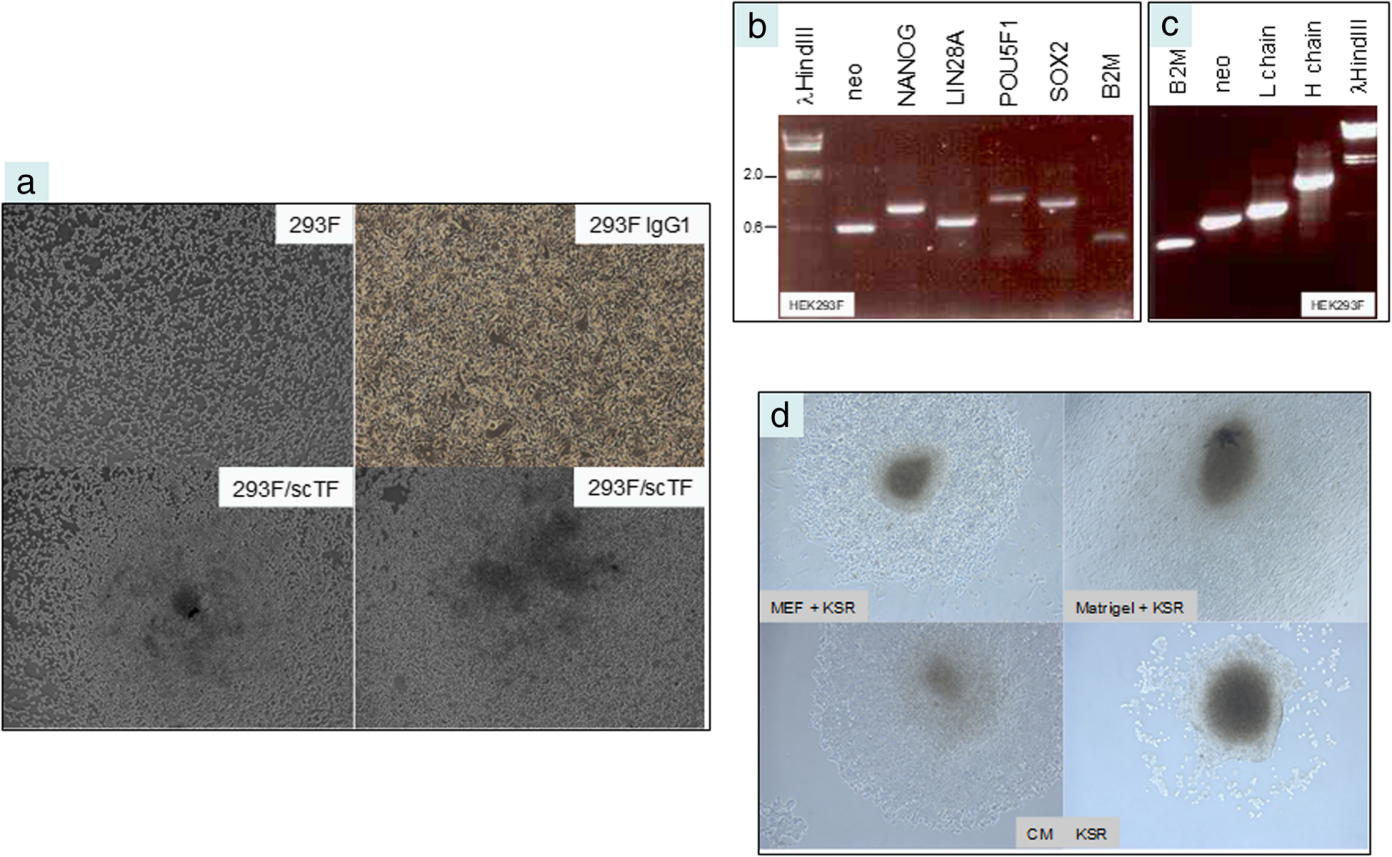
Transfected 293F cells. **a** The photomicrographs show confluent untransfected 293F cells, confluent 293F transfected with an IgG1 plasmid, 293F cells transfected with scTF plasmids. Magnification 50X. **b** Gene expression of scTF-transfected 293F* cells: 630 bp LIN28A, 930 bp NANOG, 1100 bp POU5F1, 960 bp SOX2 (size difference to the corresponding ones in Fig. 1 is due to different primers). **c** Gene expression of IgG-transfected 293F cells (720 bp L chain; 1420 bp H chain). The level of B2M in scTF-transfected cells is lower than that in IgG1-transfected cells (compared to those of neo). **d** Shown are 293F* colonies on different culture media formulations: MEF + KSR, Matrigel + KSR, CM, KSR. Magnification 50X

Both supercoiled and *Pac*I-linearized pLP4 and pSN2 were equally effective in transfection by electroporation. Figure 2a shows two resultant neo^R^ 293F* (scTF-transfected 293F) colonies with cells accumulating in the middle of each colony. This morphological appearance was not seen in untransfected 293F nor 293F/IgG1 transfected with pVITRO1 containing human immunoglobulin heavy and light chain gene modules (Fig. 2a). In our transformation procedure, no additional promoting agents like polybrene, histone deacetylase inhibitors Na butyrate and suberoylanilide hydroamic acid, nor MEF and hypoxia were included [10, 18]. Gene expression analysis of 293F* cells in CM showed the presence of full length LIN28A, NANOG, POU5F1, SOX2, plus neo mRNA (Fig. 2b). B2M expression was down-regulated when compared with that in cells transfected with immunoglobulin genes (Fig. 2c). The equivalent intensity of the neo product provided an internal control. DNA microarray analysis of LNCaP* corroborated the B2M result (see below). In these experiments, the transformed cells incorporated both pLP4 and pSN2 so that plasmids with different drug markers were not necessary.

The cloned 293F* cells were grown under different conditions. In CM and normoxia, 293F* colonies displayed the rounded appearance of stem cell colonies (Fig. 2d). In serum-free media and hypoxia, the cells became detached from the plastic surface like parental 293F cells under serum-free condition. The attached colony morphology was regained in KSR + MEF and hypoxia (with underlying MEF cells) and KSR + Matrigel and hypoxia. There was no gross difference between cells grown under hypoxia vs. normoxia. The level of expression was equivalent among the five transgenes: neo, LIN28A, NANOG, POU5F1, SOX2. The emergence of 293F* cells indicated that the cancer cell-derived scTF were fully functional in reprogramming.

### Functional testing of LuCaP 145.1-derived scTF genes in reprogramming of prostate cancer cells

**Fig. 3:**
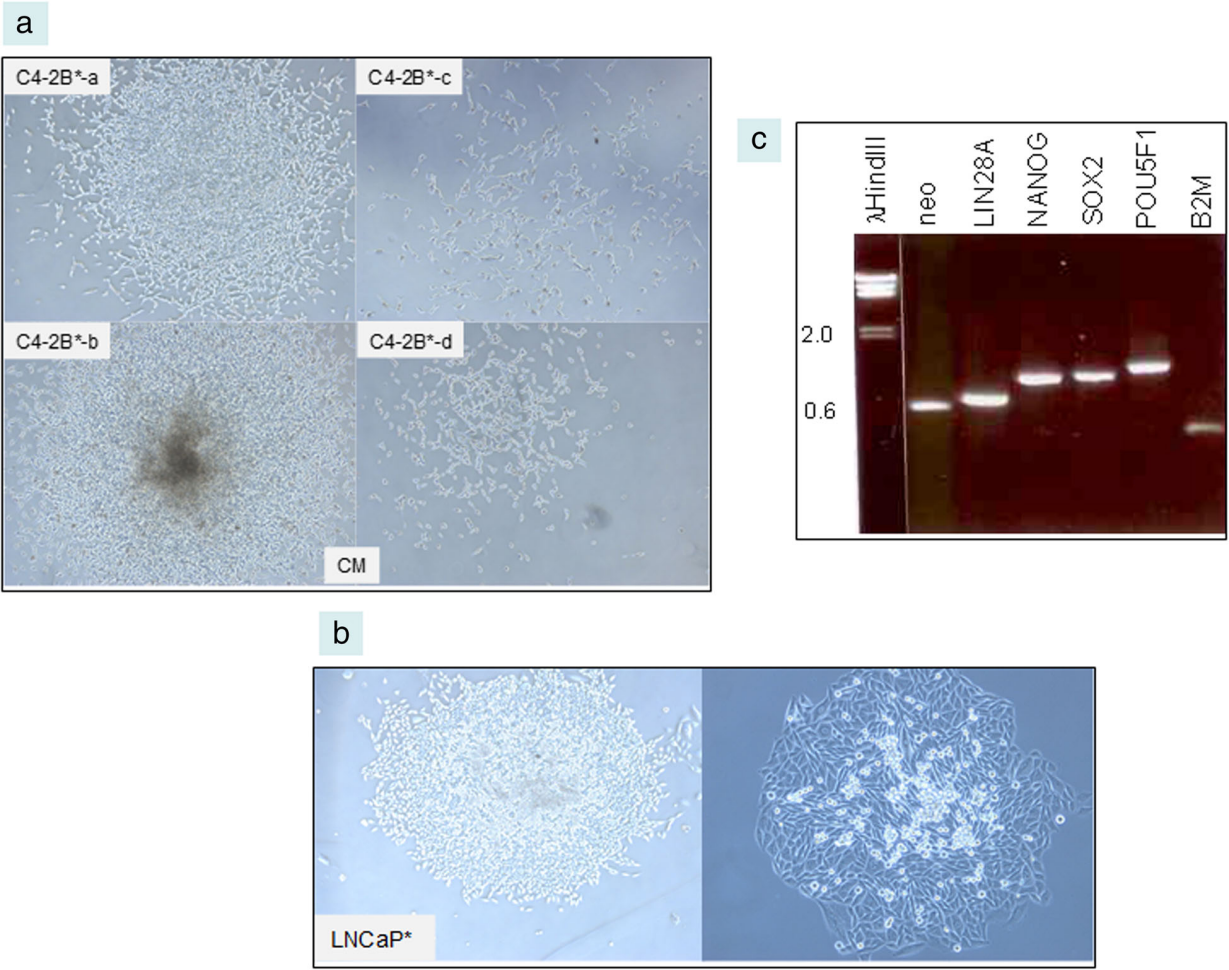
Transected C4–2B and LNCaP cells. **a** The photomicrographs show C4–2B* cells growing in four areas of the plate in CM. Magnification: 50X. **b** Two colonies of LNCaP* cells are shown (magnification: 50X left and 100X right). This colony morphology is not seen for untransfected C4–2B or LNCaP cells. **c** The gel electrophoregram shows scTF, neo and B2M detected by RT-PCR in LNCaP* grown in CM

Three prostate cancer cell lines were transfected by the scTF plasmids. G418 selection allowed transfected cells to grow out. The neo^R^ colonies of C4–2B* appeared similar to those of 293F* in CM (Fig. 3a). The photomicrographs show four areas of the C4–2B* plate, which could represent stages of proliferation towards colony formation. C4–2B* cells could also be grown in KSR + MEF and hypoxia. Gene expression analysis of C4–2B* in CM revealed the presence of the scTF plus down-regulation of B2M. Dataset query of archived cell-type transcriptome datasets showed absence of these genes in the prostate cancer cell lines used, CD26^+^ Gleason pattern 3 cancer, as well as differentiated prostate CD26^+^ luminal, CD49a^+^ stromal, and CD104^+^ basal cells. For B2M, the array signal intensity values for cancer cell lines averaged at 5500 vs. 1600 for stem cells. Differentiated cell types had an average value of 12,000.

Transfected LNCaP* cells displayed a similar colony morphology (Fig. 3b). Expression analysis showed the presence of the four scTF and lower B2M (Fig. 3c). Under high power, LNCaP* and C4–2B* cells at the colony periphery appeared dissimilar. After trypsin treatment and passaging in CM, individual LNCaP* and C4–2B* cells displayed a different morphology with C4–2B* cells forming a network-like structure and cells sprouting slender processes. LNCaP* colonies were more compact. FBS in CM is known to induce undirected differentiation of stem cells [22].

**Fig. 4:**
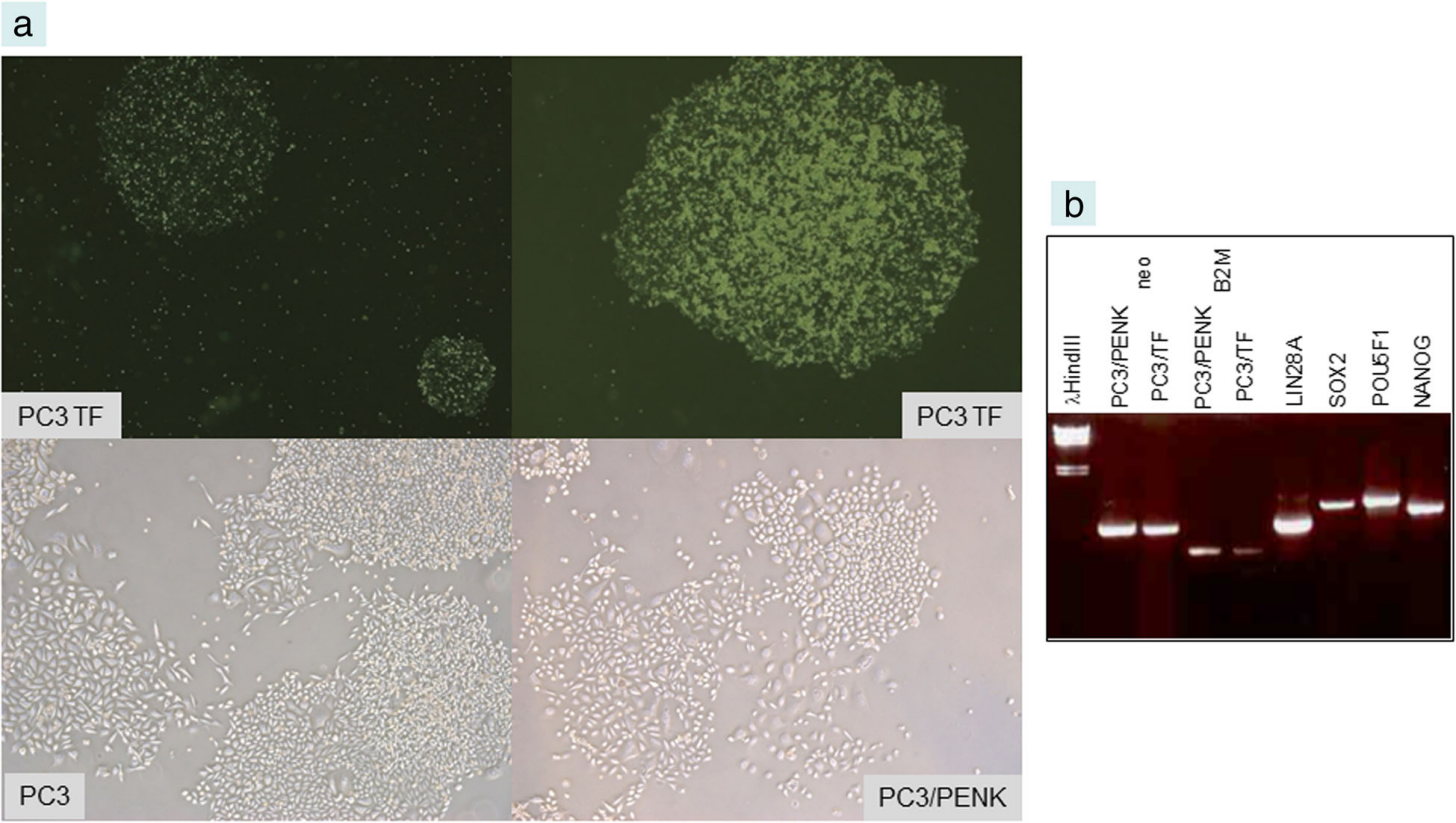
Transfected PC3 cells. **a** PC3* cells also grew in rounded colony morphology. Magnification 25X. For comparison, untransfected PC3 and PC3 transfected with PENK are included. Magnification 50X. **b** Downregulation of B2M is also seen in PC3/scTF vs. PC3/PENK (compared to those of neo)

PC3, unlike LNCaP and C4–2B, is more stem-like by its transcriptome [2]. The colonies of PC3* also displayed the rounded morphology (Fig. 4a) and downregulation of B2M (Fig. 4b). The colony morphology was different from that of PC3 or PC3 transfected with a non-scTF gene PENK (Fig. 4a).

### Link between B2M and scTF expression

**Fig. 5:**
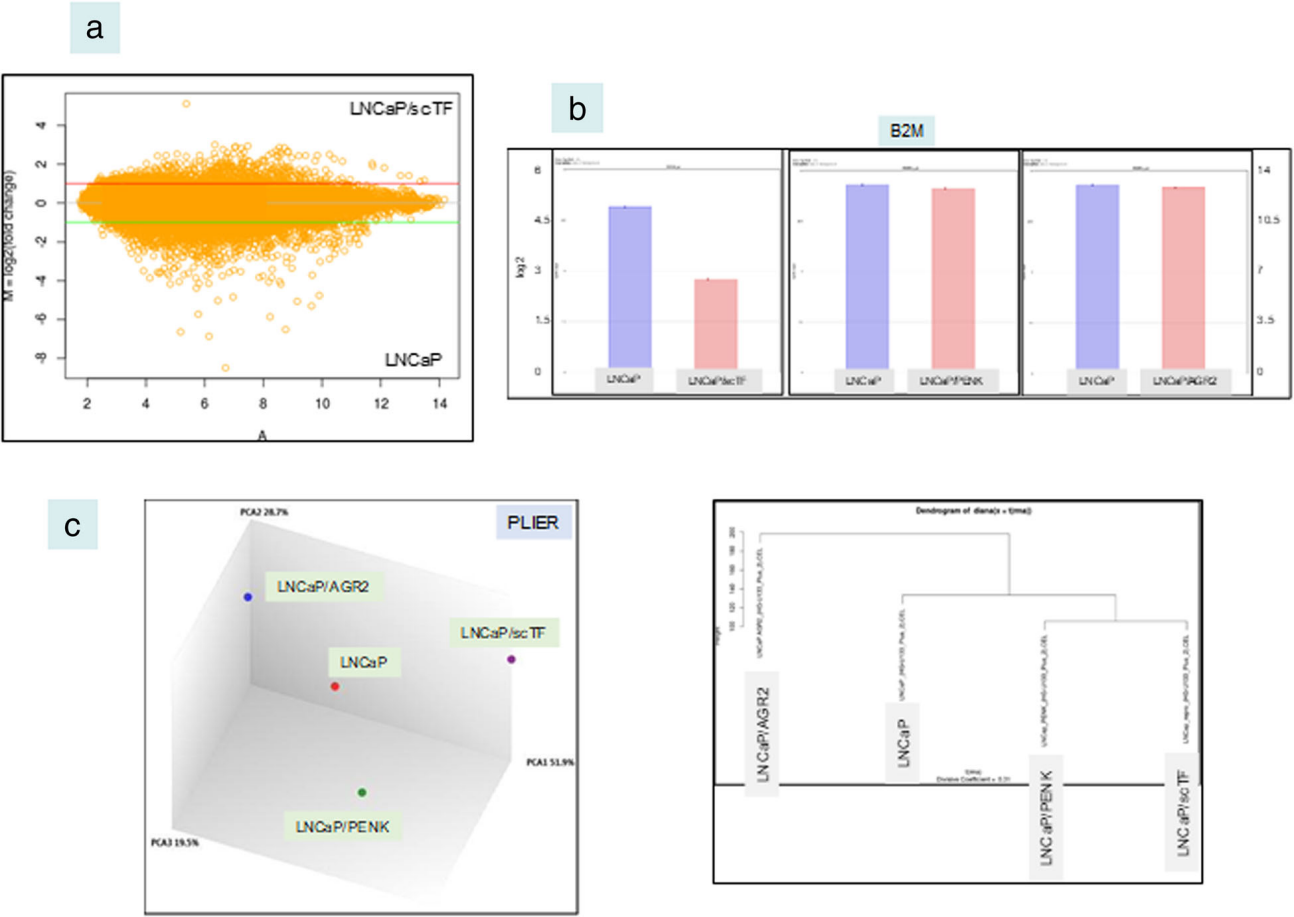
DNA microarray data analysis. **a** Expression difference between LNCaP* and LNCaP is visualized by the MA plot. **b** Based on array signal intensity values, B2M level is lower in LNCaP* compared with LNCaP, LNCaP/PENK and LNCaP/AGR2. Signal values in log_2_ are indicated on the *y*-axis. The second and third panels have a different set of values on the *y*-axis than the first panel. **c** The PCA plot of the four LNCaP datasets shows the difference in the transcriptomes of the four cell types. PLIER (probe logarithmic intensity error estimation) is a multi-array normalization method to produce improved signals by accounting for experimentally observed patterns in feature behavior, and handling error at low and high signal values. A dendrogram depiction of the data is also shown

DNA microarrays were used to compare the transcriptomes of LNCaP* and parental LNCaP. The MA plot showed gene expression changes upon scTF transfection (Fig. 5a). The decrease in B2M signal intensity level was confirmed. For comparison, no decrease was found in LNCaP cells transfected with either anterior gradient 2 (AGR2) or proenkephalin (PENK) (Fig. 5b, and Additional file 1). AGR2 is an adenocarcinoma gene upregulated in primary prostate cancer cells [3], while PENK is a prostate stromal cell-specific gene absent in tumors [23]; both are candidate signaling molecules in prostate stromal/epithelial interaction. Gene expression changes in LNCaP/PENK and LNCaP/AGR2 are shown in a PCA plot of the array datasets (Fig. 5c). The expression differences also indicated that the transfected genes were translated into their respective functional proteins in mediating these changes. Secreted AGR2 was detected by ELISA [24] in the culture media of LNCaP/AGR2.

**Fig. 6:**
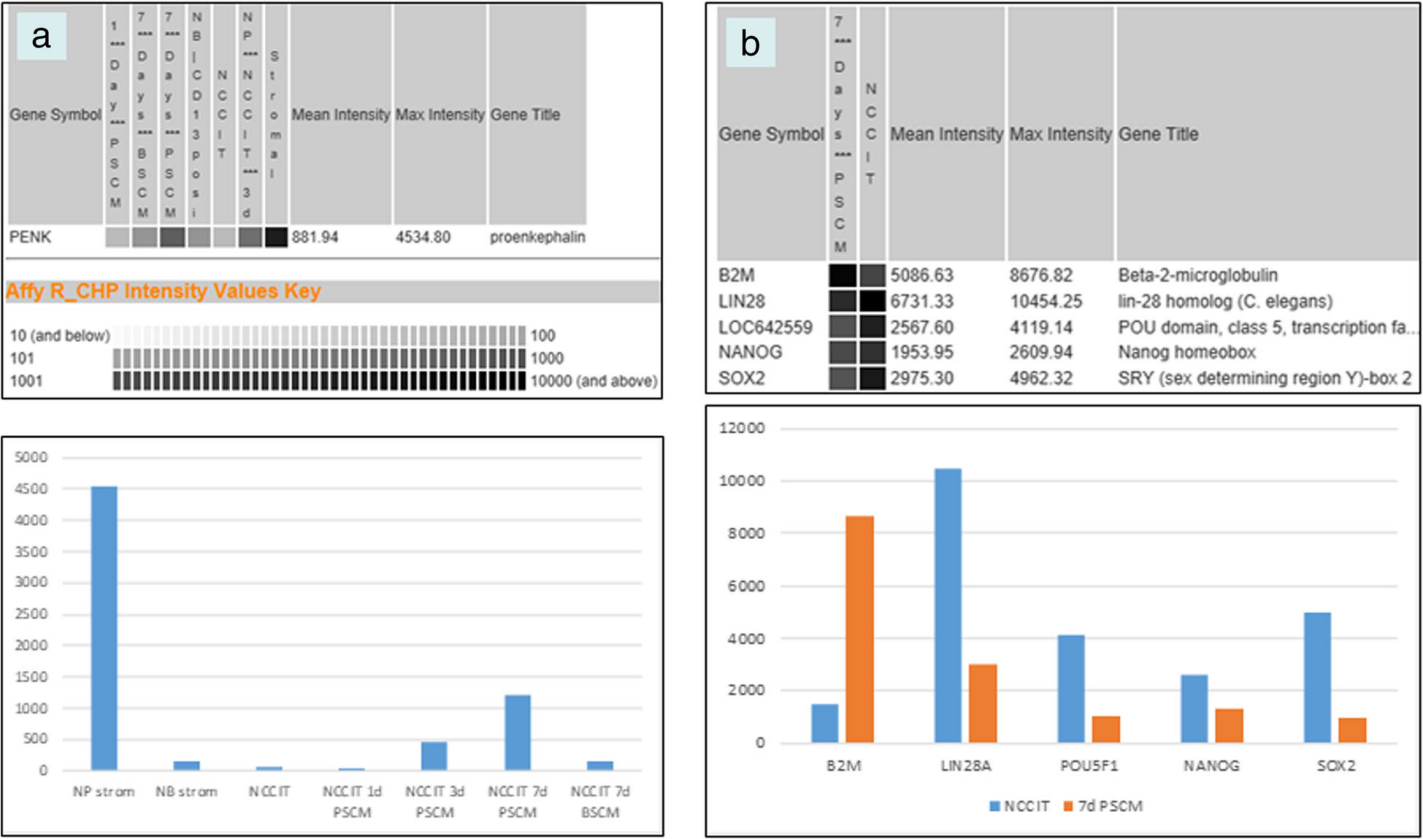
Dataset query. Array probeset signal values are shown on gray scale and histogram format (*y*-axis). **a** PENK levels in treated NCCIT cells: NP strom = CD49a^+^ prostate stromal cells, NB strom = CD13^+^ bladder stromal cells, PSCM = prostate stromal conditioned media, BSCM = bladder stromal conditioned media. Time points are indicated on the *x*-axis. **b** scTF and B2M levels in NCCIT and at 7d PSCM. LOC642559 is the array probeset for POU5F1. Transcriptome data were queried from three replicates of NCCIT and four replicates of NCCIT + PSCM

The differential expression of B2M was supported by dataset query of induction of stem cell differentiation by stromal cell factors [4]. The EC cell line NCCIT is stem-like with similar gene expression as ES cells [18]. NCCIT cells were incubated with stromal cells across a semipermeable membrane. At various time points, the treated NCCIT cells were analyzed by DNA microarrays. PENK was found differentially expressed between prostate and bladder stromal cells (as shown in Fig. 6a, histogram bars 1 and 2) [23]. The PENK level in NCCIT was increased from d1 to d7 with prostate stromal induction, which was not observed with bladder stromal induction (Fig. 6a, bars 4–6 vs. 7). Concurrently, the four scTF genes in NCCIT showed a decrease in signal values while B2M showed an increase (Fig. 6b), the opposite in reprogramming.

## Discussion

Small cell carcinoma is a rare but lethal form of prostate cancer comprising 5% of cancers [25]. The presence of prostate cancer-specific TMPRSS2-ERG fusion in both adenocarcinoma and small cell carcinoma of the same tumor cases suggests a direct lineage [26]. In one way, prostate small cell carcinoma with neuroendocrine differentiation is regarded as trans-differentiation of adenocarcinoma based on research using LNCaP [27]. In our research, the transcriptome of small cell carcinoma LuCaP 145.1 was found to be closest to that of ES than those of other prostate cancer cell types - cell lines, cells isolated from primary tumors, and adenocarcinoma LuCaP lines. The ES proximity was confirmed by the expression of LIN28A, NANOG, POU5F1 and SOX2 in LuCaP 145.1. The newly available LuCaP 93 small cell carcinoma was found to express these scTF, but with lower NANOG. The strength of NANOG expression could affect the conversion of cancer cell density from [epi] to [strom]. LuCaP 145.1, as indicated by banding at [strom], had completely lost its epithelial characteristics (i.e., like stem cells). The other tumors still contained cells banding at both [epi] and [strom]. The difference in gene expression among the LuCaP small cell carcinoma lines reflected the finding of multiple small cell carcinoma subtypes in human tumors [28]. Expression heterogeneity was also found among LuCaP adenocarcinoma lines regarding the scTF genes – many with POU5F1, a few with LIN28A, none with SOX2 and NANOG [2, 10]. The aggressive behavior and therapy resistance of prostate small cell carcinoma could be attributed to their stem-likeness, because stem cells are equipped to survive over an organism’s lifespan. Reports in the literature have documented the association between LIN28 [29], NANOG [30], POU5F1 [31], and SOX2 [32] and prostate cancer aggressiveness individually.

Our experiments also tried to determine if the scTF in LuCaP 145.1 were responsible for its stem-like expression. These genes were cloned for reprogramming testing. Transfection of 293F fibroblasts as well as prostate cancer cells LNCaP, C4–2B, and PC3 produced cells with stem-like colony morphology and down-regulated B2M, indicating that the proteins encoded by these genes were functional. Their functionality is equivalent to that of the same scTF cloned from ES cells being used to reprogram somatic cells [11], prostate cancer-associated stromal cells [17], and LuCaP adenocarcinoma lines [10]. The transformed cells could be propagated in serum-free media under hypoxia, which could inhibit cells like parental LNCaP [33]. Furthermore, increase in B2M expression is associated with differentiation while decrease with de-differentiation as exhibited by LuCaP 145.1.

We postulate that the transition of prostate cancer from adenocarcinoma to non-adenocarcinoma and small cell carcinoma involves activation of scTF genes in the sequence of POU5F1 → LIN28A/SOX2 → NANOG with tumor cells adopting a more de-differentiated state. SOX2, for example, is found in the undifferentiated developing prostate [32], and is responsible for the maintenance of neural progenitors [34]. Our proposed scheme of prostate cancer de-differentiation could proceed from Gleason pattern 3 adenocarcinoma/LNCaP → C4–2B → Gleason pattern 4 → PC3/non-adenocarcinoma (LuCaP 173)/SOX2^+^ small cell carcinoma LuCaP 49 [2] → LuCaP 93 → LuCaP 145. It would be interesting to compare prostate small cell carcinoma with small cell carcinoma of lung and bladder, which have recently been analyzed by exome sequencing [35]. The exome sequencing data revealed no single thematic pattern for these small cell carcinoma such as a high number of DNA mutations, which is similar to what was found by exome sequencing of LuCaP small cell carcinoma and LuCaP adenocarcinoma [12]. The poorly differentiated prostate small cell carcinoma phenotype is, at least, not due to an accumulation of genomic changes over time. We could explore whether the four scTF are expressed by these other small cell carcinoma types, whether reprogrammed non-small cell lung or urothelial cancer cells show similar expression as reprogrammed prostate cancer cells. We hypothesize that stem-like prostate cancer cells may also respond to stromal cell signaling as shown by the germ cell tumor-derived NCCIT.

The advantage afforded by our plasmid vectors includes biosafety over the previously used lentiviral vectors, especially since these scTF genes could be potentially oncogenic (http://cancer.sanger.ac.uk/cosmic/census/tables?name=symbol) due to their expression in cancer. Ample plasmid DNA could be obtained from small cultures while lentiviral vectors require expertise, high cost and a complicated procedure to produce transfection-ready stocks [18]. Other commercially available viral vectors, e.g., CytoTune Sendai virus [36], are also expensive for relatively small amounts of DNA. We have also tried other plasmid-based vectors [37] in our earlier studies, but found very low transformation efficiency due perhaps to the need for co-transfection of several separate plasmids. The drug selection marker (neo) allows transformation of fast growing cancer cell lines in which untransfected cells would otherwise overwhelm transfected cells without it. Additionally, vectors containing antisense scTF genes (cloned in the 3′ → 5′ orientation) can be used to inactivate the genes in LuCaP 145.1, for example, to determine if forced differentiation could lead to an adenocarcinoma-like derivative.

## Conclusions

Prostate small cell carcinoma exhibits characteristics of stem cells, including poor differentiation (e.g., loss of epithelial cell density, non-luminal-like) and lower B2M expression due to the reactivation of stem cell transcription factors.

## Additional files

Additional file 1: Plasmid vector construction (DOCX 13 kb)

Additional file 2: **Figure S1.** scTF plasmid vectors. **a** Full-length RT-PCR products of the scTF genes were obtained from LuCaP 145.1. **b** The schematics of plasmids pLP4 and pSN2 (and symbols for control elements of gene expression) are shown. The *Pac*I sites are used for linearization. (TIF 25138 kb)

## Abbreviations

[epi]: epithelial cell density
[strom]: stromal cell density
AGR2: anterior gradient 2
bp: base pairs
CM: complete media
ELISA: enzyme-linked immuosorbent assay
ES: embryonic stem
FBS: fetal bovine serum
HEK293F: human embryonic kidney fibroblast
IgG1: immunoglobulin 1
iPS: induced pluripotent stem
KSR: KnockOut Serum Replacement
LB: Luria broth
MEF: mouse embryo fibroblast
NCCIT: embryonal carcinoma cell line
neo: neomycin
PCA: principal components analysis
PDX: patient-derived xenograft
PENK: proenkephalin
PSA: prostate- specific antigen
RT-PCR: reverse transcriptase-polymerase chain reaction
SCID: severe combined immunodeficient mice
scTF: stem-cell transcription factors
TURP: transurethral resection of the prostate
