# Supplementary material for: Lineage relationship between prostate adenocarcinoma and small cell carcinoma
Source: BMC Cancer. 2019 May 30;19:518. doi: 10.1186/s12885-019-5680-7 (PMC6543672; doi:10.1186/s12885-019-5680-7)
Supplement: Supplementary file 1 — Plasmid vector construction (DOCX 13 kb) [file 12885_2019_5680_MOESM1_ESM.docx]

**Supplementary Materials**

**Plasmid vector construction**

RT-PCR with primers encompassing the coding regions was used for cloning into plasmid pVITRO1-neo (InvivoGen, San Diego, CA). The primer pairs were NANOG-5EcoRV gggccc*gataTC*TATACTAACATGAGTGTGGA**C**CCAGCTTGTCCCCAAAG (non-genic sequences in lower case, restriction enzyme sequences for cloning in italics; start codon ATG or stop codon TCA (antisense) underlined; C in bold is used instead of T to remove a gene-internal *Bam*HI site) and NANOG-3BamHI gctga*ggatcc*TCACACGTCTTCAGGTTGCATGTTC; LIN28A-5BamHI gctga*ggatcC*AGCCGACGACCATGGGCTCCGTGTCC and LIN28A-3AvrII tccgaattc*cctagg*TCAAT

TCTGTGCCTCCGGGAGCAGG; POU5F1-5EcoRV tcagggccc*gatatC*TTCCTTCCCCATGGCGGGACA

CCTG and POU5F1-3BamHI gctga*ggatCC*TCAGTTTGAATGCATGGGAGAGCCC; SOX2-5BamHI gctga*ggatcC*AGCGCCCGCATGTACAACATGATGG and SOX2-3AvrII tccgaattc*cctagg*TCACATGT

GTGAGAGGGGCAGTGTG. About ten nucleotides upstream of ATG were included to retain the Kozak box. The expected product sizes were 630 bp LIN28A, 930 bp NANOG, 960 bp SOX2, 1100 bp POU5F1 (Additional file Figure S1a). The PCR products were purified (Qiagen QIAquick, Germantown, MD), and digested by either *Eco*RV/*Bam*HI for the POU5F1 and NANOG modules or *Bam*HI/*Avr*II for the LIN28A and SOX2 modules. These were ligated to *Eco*RV/*Bgl*II or *Bam*HI/*Avr*II-digested, calf intestinal phosphatase-treated pVITRO1. Plasmid pLP4 (8.03 kb) was obtained by first insertion of the LIN28A module followed by that of the POU5F1 module; plasmid pSN2 (8.19 kb) was obtained by first insertion of the SOX2 module followed by that of the NANOG module (Additional file Figure S1b). Bacterial colonies were screened by PCR and restriction enzyme digestion. Plasmids containing only one gene were also generated: pLIN28A, pNANOG, pPOU5F1, pSOX2. The plasmid constructs were transfected into cold CaCl_2_-treated TOPO1 bacterial cells (ThermoFisher). Kanamycin (neo, InvivoGen)-resistant bacterial colonies were screened by PCR, and positive clones were grown in 1 ml LB media overnight. Plasmid DNA was isolated using Qiagen miniprep kit, and the vectors were linearized by digestion with *Pac*I.

For PENK vector, oligonucleotide pairs pK1EcoRV cagggccc*gatatc*GCGTCAACTCCATGGCG

CGGTTCC and pK3BamHI gctga*ggatcc*ATTAAAATCTCATAAATCCTCCGTTCTTTTTTC; pK2BamHI gctga*ggatcc*GCGTCAACTCCATGGCGCGGTTCC and pK4AvrII tccgaattc*cctagg*ATT

AAAATCTCATAAATCCTCCGTATCTTTTTTC were used to amplify an 803-bp PENK-encoding gene module from cDNA generated from dissected benign prostate tissue [23]. The products were digested with *Eco*RV/*Bam*HI and *Avr*II/*Bam*HI, respectively, and ligated into pVITRO1 to produce a vector containing two expression cassettes of PENK. Plasmid pK^2^-3 was used for transfection. For AGR2 (anterior gradient 2) vector, oligonucleotide pair AGR2-5BamHI gctga*ggatcC*ACAAGGCAGAGTTGCCATGGAG and AGR2-3AvrII tccgaatt*cctagg*TTACAATTCAG

TCTTCAGCAACTTG were used to amplify a 544-bp AGR2-encoding gene module from prostate cancer specimens 04-176C and 04-184C [3]. The product was digested with *Bam*HI/*Avr*II for ligation into pVITRO1 to generate pAGR2-1.
